# Dexmedetomidine-treated hyperventilation syndrome triggered by the distress related with a urinary catheter after general anesthesia: a case report

**DOI:** 10.1186/s40981-017-0101-x

**Published:** 2017-05-08

**Authors:** Junichi Saito, Erika Amanai, Kazuyoshi Hirota

**Affiliations:** Department of Anesthesiology, Hirosaki University Graduate School of Medicine, Zaifu-cho 5, Hirosaki, 036-8562 Japan

**Keywords:** Hyperventilation syndrome, Dexmedetomidine, General anesthesia

## Abstract

**Background:**

Hyperventilation syndrome (HVS) sometimes occurs in patients under stressful conditions and may provoke severe complications such as myocardial infarction and death. The authors report a case of HVS following general anesthesia, where a continuous intravenous infusion of dexmedetomidine was effective for HVS.

**Case presentation:**

A 23-year-old male patient with recurrent tongue cancer was scheduled to undergo partial glossectomy and neck dissection. Emergence from general anesthesia was prompt. Twenty-two minutes after extubation, the patient complained of unbearable distress caused by the urinary catheter. He began to cry, with an increased respiratory rate of over 40 breaths per minute. Intravenous infusion of flurbiprofen, droperidol, and morphine was not effective. Electrocardiography and laryngofiberscopy indicated the absence of acute coronary syndrome and airway obstruction, respectively. An arterial blood gas determination showed pH 7.63, PaCO_2_ 18.2 mmHg, PaO_2_ 143 mmHg on O_2_ mask 4 L/min, Ca^2+^ 4.29 mmol/L, and lactate 3.4 mmol/L. The patient was diagnosed with HVS. Dexmedetomidine infusion 2.0 μg/kg/h for 10 min followed by 0.7 μg/kg/h reduced respiratory rate, suppressed arousal, and disappeared the complaint of bladder distension. One hour after extubation, an arterial blood gas determination showed pH 7.33, PaCO_2_ 51.3 mmHg, PaO_2_ 196 mmHg on O_2_ mask 4 L/min, Ca^2+^ 4.70 mmol/L, and lactate 1.5 mmol/L. After admission to the intensive care unit, dexmedetomidine infusion was maintained at the rate of 0.2 to 0.7 μg/kg/h until the following morning, and he did not complain of distress caused by the urinary catheter.

**Conclusions:**

HVS can occur after emergence from general anesthesia, and dexmedetomidine infusion was effective for HVS.

## Background

Hyperventilation syndrome (HVS) may occur in relatively young patients under a stressful condition. HVS is usually considered to be benign; however, hyperventilation and subsequent respiratory alkalosis may provoke severe complications such as myocardial infarction and death [1–4]. Thus, early diagnosis and treatment is essential to prevent these complications. The authors report a case of HVS triggered by distress caused by the urinary catheter after otolaryngologic surgery. Continuous intravenous infusion of dexmedetomidine was effective for hyperventilation and control of symptoms.

## Case presentation

A 23-year-old male patient with recurrent tongue cancer was scheduled to undergo partial glossectomy and neck dissection. His height and body weight were 171 cm and 76.1 kg, respectively. He had no history of psychiatric disease or HVS. Throughout the general anesthesia, the following variables were monitored continuously: electrocardiogram (ECG), peripheral oxygen saturation (SpO_2_), end-tidal concentration of carbon dioxide. left radial artery pressure, rectal temperature, and bispectral index. Anesthesia was induced and maintained with propofol (3–6 mg/kg/h), ketamine (70 mg), remifentanil (0.15–0.5 μg/kg/min), and rocuronium (50 mg). To reduce postoperative pain, fentanyl 200 μg and acetaminophen 1000 mg were intravenously administered 30 min before the end of the surgery. Emergence from general anesthesia was prompt, and the trachea was extubated. An initial arterial blood gas determination showed pH 7.38, PaCO_2_ 45.2 mmHg, PaO_2_ 193 mmHg on O_2_ mask 4 L/min, Ca^2+^ 4.70 mmol/L, and lactate 1.3 mmol/L 10 min after extubation. Twenty-two minutes after extubation, the patient complained of unbearable distress caused by the urinary catheter. Though flurbiprofen axetil 50 mg was intravenously administered, the symptom did not improve but gradually increased. The patient began to cry and his voice was hoarse, with an increased respiratory rate over 40 breaths per minute. The ECG revealed normal sinus tachycardia (HR 100–120 bpm) without ST segment changes. A laryngofiberscopy indicated an absence of vocal fold paralysis and laryngeal edema. The patient was diagnosed with HVS. Although droperidol 5 mg and morphine 10 mg were intravenously administered, hyperventilation did not improve. An arterial blood gas determination showed pH 7.63, PaCO_2_ 18.2 mmHg, PaO_2_ 143 mmHg on O_2_ mask 4 L/min, Ca^2+^ 4.29 mmol/L, and lactate 3.4 mmol/L. Thirty-five minutes after extubation, continuous sedation with dexmedetomidine was selected to treat hyperventilation and symptoms. Dexmedetomidine infusion 2.0 μg/kg/h for 10 min followed by 0.7 μg/kg/h reduced respiratory rate, suppressed arousal, and disappeared the complaint of bladder distension. Even though apnea (≦4 breaths per minute) after hyperventilation appeared for 3 min, SpO_2_ was maintained at 90% or above. One hour after extubation, arterial blood gas analysis showed pH 7.33, PaCO_2_ 51.3 mmHg, PaO_2_ 196 mmHg on O_2_ mask 4 L/min, Ca^2+^ 4.70 mmol/L, and lactate 1.5 mmol/L. After admission to the intensive care unit, dexmedetomidine infusion was maintained at the rate of 0.2 to 0.7 μg/kg/h until the following morning, and he did not complain of distress caused by the urinary catheter.

## Discussion

This case provided two important clinical issues. First, HVS can occur after emergence from general anesthesia. This syndrome is a common disease as indicated in a study by Jones et al., estimating a prevalence of 9.5% in an adult population [5] and sometimes occurring during dental care [6, 7]. However, HVS after general anesthesia is less common because sedative drugs and opioids provide an anxiolytic and antiadrenergic action and relief of pain. Mizuno and colleagues [8] reported a case in which HVS developed before induction of and after emergence from general anesthesia. The authors considered that this syndrome could occur repeatedly under anxiety and stressful conditions even if a patient had no previous history of psychiatric disease. In this case, potential anxiety about tongue cancer induced HVS triggered by bladder distension. Anesthesiologists should consider that HVS can occur even after general anesthesia.

Although HVS is a common disease, HVS is a disorder with no widely accepted diagnostic criteria [9]. Thus, it is diagnosed by the exclusion of alternative possibilities such as metabolic and cardiopulmonary diseases, asthma, pneumothorax, airway obstruction, and acute coronary syndrome. In this case, ECG, laryngofiberscopy, and arterial blood gas analysis ruled out these diseases and he was diagnosed with HVS. However, delirium or agitation also occurs after general anesthesia and differential diagnosis between them is difficult, and the overlap of delirium or agitation that are associated with hyperventilation should be kept in mind. Delirium is associated with increased mortality, prolonged hospital stay, and persistent cognitive dysfunction [10, 11]. Early diagnosis and treatment for altered mental status occurring in the postoperative period after emergence from general anesthesia are important.

Second, dexmedetomidine infusion was effective for HVS. Dexmedetomidine is an α-adrenergic agonist with dose-dependent α_2_-adrenoceptor selectivity. It seems that the mechanism of action of the drug is suitable to treat HVS. Pre- and post-synaptic activation of α_2_-adrenoceptor inhibits the release of norepinephrine and sympathetic activity and terminates the transmission of pain signals [12]. These effects produce sedation, analgesia, and anxiolysis. Furthermore, α_2_-adrenoceptor inhibits the release of serotonin in the rostral raphe nuclei which is associated with arousal and fear which are associated with panic disorder or others [13]. A large randomized control study revealed that dexmedetomidine reduced the incidence of postoperative delirium by 14% in patients aged over 65 years [14]. In addition to analgesic and anxiolytic properties of dexmedetomidine, its anti-inflammatory action might have treated both HVS and delirium. Elevated biomarkers of inflammation have associated with the risk for developing delirium [15]. Anti-inflammatory effects of dexmedetomidine were evident in both clinical and animal studies [16, 17]. Furthermore, dexmedetomidine has advantages over other sedating agents because of minimal suppression of respiratory function [18]. In this case, the bladder might have been kept distended until the next morning and upper airway obstruction and respiratory depression after neck dissection were one of several concerns. Taking these factors into consideration, the drug may be considered to be a good choice for sedation for HVS after general anesthesia. Dexmedetomidine can be an option to treat and prevent an altered mental state occurring in the postoperative period after emergence from general anesthesia.

## Conclusions

HVS can occur after emergence from general anesthesia, and dexmedetomidine infusion was effective for hyperventilation syndrome. Clinical studies and case report accumulation are required to confirm the usefulness of dexmedetomidine for HVS after general anesthesia.

## Abbreviation

HVS: Hyperventilation syndrome
